# SMYD2 in Hepatocellular Carcinoma: An Emerging Epigenetic Link Between Tumor Cell Programs and Microenvironmental Dynamics

**DOI:** 10.3390/cancers18213586

**Published:** 2026-11-08

**Authors:** Camila Galeota Satini, Barbara Bueloni, Nicolás Marcolini, Esteban Fiore, Juan Bayo, Guillermo Mazzolini

**Affiliations:** 1 Hepatology and Gene Therapy Program, Instituto de Investigaciones en Medicina Traslacional, CONICET-Universidad Austral, Av. Pte. Perón 1500, Pilar B1629AHJ, Buenos Aires, Argentina; cgaleota-iimt@austral.edu.ar (C.G.S.); bbueloni-conicet@austral.edu.ar (B.B.); nmarcoli@cas.austral.edu.ar (N.M.); efiore@austral.edu.ar (E.F.); 2 Facultad de Ciencias Biomédicas, Universidad Austral, Av. Pte. Perón 1500, Pilar B1629AHJ, Buenos Aires, Argentina; 3 Consejo Nacional de Investigaciones Científicas y Técnicas (CONICET), Av. Rivadavia 1917, Buenos Aires C1033AAJ, Argentina; 4 Spectrum Regenerative (HZ4 Liver Inc.), Miami, FL 33130, USA; 5 Oncology Service, Hospital Universitario Austral, Universidad Austral, Av. Pte. Perón 1500, Pilar B1629AHJ, Buenos Aires, Argentina; 6 Liver Unit, Hospital Universitario Austral, Universidad Austral, Av. Pte. Perón 1500, Pilar B1629AHJ, Buenos Aires, Argentina

**Keywords:** SMYD2, hepatocellular carcinoma, lysine methyltransferase, tumor microenvironment, immune evasion, metabolic reprogramming, SMYD2 inhibition

## Abstract

Hepatocellular carcinoma (HCC) usually arises within chronically injured liver tissue, where inflammation, fibrosis, metabolic dysfunction, and immune remodeling shape tumor evolution and therapeutic response. This biological complexity has increased interest in regulators capable of connecting tumor-intrinsic programs with the diseased hepatic microenvironment. SET and MYND domain-containing protein 2 (SMYD2) is a lysine methyltransferase that modifies histone and non-histone substrates involved in transcriptional control, tumor suppressor regulation, DNA damage repair, metabolism, cellular plasticity, and inflammatory signaling. In HCC, increased SMYD2 expression has been associated with aggressive clinicopathological features and poor outcome, while functional studies link SMYD2 to tumor cell proliferation, c-Myc/GLS1-dependent glutamine metabolism, stemness-associated programs, sorafenib resistance, and immune evasion. Mechanistically, SMYD2 may act through multiple context-dependent pathways, including β-catenin methylation, BMP4/SMAD/ID3 signaling, TLR4/NF-κB-dependent hepatic stellate cell activation, and Wnt ligand-mediated tumor–macrophage communication, leading to TGF-β-associated immune suppression. In preclinical HCC models, SMYD2 inhibition has reduced tumor growth and enhanced responses to sorafenib or anti-PD-1 treatment, although its clinical efficacy and safety remain to be established. This translational uncertainty is particularly relevant because SMYD2 dependency is not uniform across cancer models, and its functions in non-tumoral liver, macrophages, and acute hepatic injury suggest that systemic inhibition may have compartment- and context-specific consequences. This review summarizes the structural basis, biological functions, and therapeutic implications of SMYD2 in HCC, emphasizing both its mechanistic relevance and the questions that will need to be resolved before clinical translation.

## Simple Summary

Hepatocellular carcinoma, the most common form of liver cancer, often develops in a liver affected by long-term inflammation, scarring, and metabolic disease. This review examines SMYD2, a protein that modifies other proteins and can influence both cancer cells and the surrounding liver tissue. Research links SMYD2 to tumor growth, changes in how cancer cells use nutrients, stem-like properties, resistance to treatment, and interactions with immune cells. Additionally, in preclinical HCC models, blocking SMYD2 has reduced tumor growth and improved responses to anti-PD-1 treatment. However, SMYD2 also influences noncancerous liver tissue and immune cells, raising concerns about the translation of these findings into clinical practice. Further research is needed to establish the safety and efficacy of SMYD2 inhibition in patients and identify which tumors are most likely to benefit.

## 1. Introduction

Liver cancer remains a major cause of cancer-related mortality worldwide, and hepatocellular carcinoma (HCC) accounts for approximately 75–85% of primary liver cancers [1,2]. Despite advances in therapeutic strategies, its five-year survival rate remains below 20% [2]. HCC usually arises in the setting of chronic liver injury or cirrhosis, although it may also develop in non-cirrhotic livers in selected etiological contexts [3,4]. Accordingly, prognosis and therapeutic decision-making are shaped not only by tumor burden and biological aggressiveness, but also by the degree of underlying liver dysfunction. Major etiological drivers include chronic hepatitis C virus (HCV) and hepatitis B virus (HBV) infections, alcohol-associated liver disease (ALD), and metabolic dysfunction-associated steatotic liver disease (MASLD). Although these disorders differ in their initiating mechanisms, they converge on chronic injury, inflammation, fibrogenesis, metabolic dysfunction, and tissue remodeling [5]. This etiological diversity contributes to the marked heterogeneity of HCC, which extends across spatial, temporal and microenvironmental dimensions [6].

The management of HCC remains challenging despite substantial therapeutic progress [7,8]. Potentially curative approaches, including surgical resection, liver transplantation, and local ablation, are largely restricted to early-stage disease [9]. For patients with intermediate or advanced HCC, treatment selection is determined by the interaction among tumor burden, liver function, performance status, and the feasibility of locoregional or systemic therapy [7].

In recent years, multikinase inhibitors and immune checkpoint inhibitor-based combinations have improved clinical outcomes; however, durable responses remain limited to a subset of patients, and both primary and acquired resistance are common [7,10,11]. The coexistence of tumor heterogeneity and a chronically diseased hepatic microenvironment further constrains therapeutic efficacy and contributes to variable clinical responses [12]. These limitations underscore the need to identify molecular regulators that integrate oncogenic signaling with the inflammatory, fibrotic, metabolic and immune features of the diseased liver [13].

Chromatin modifiers and post-translational regulators are increasingly recognized as central components of this interface because they translate microenvironmental cues into transcriptional and signaling outputs [14,15]. Lysine methyltransferases are particularly relevant in this regard, as they modify both histone and non-histone substrates involved in tumor-suppressor control, metabolic adaptation, DNA repair, cellular plasticity, and immune regulation [16].

Among them, SET and MYND domain-containing protein 2 (SMYD2; KMT3C) has emerged as a context-dependent molecular integrator rather than a regulator of a single linear pathway [17,18]. Evidence from HCC and other tumor models links SMYD2 to oncogenic signaling, metabolic reprogramming, stem-like tumor states, treatment resistance, and communication between tumor cells and the surrounding microenvironment. This review critically examines the structural and biological properties of SMYD2, its contribution to chronic liver disease and hepatocarcinogenesis, and the opportunities and challenges associated with its therapeutic targeting in HCC.

## 2. SMYD2: Structure and Function

SMYD2, also known as KMT3C, is a lysine methyltransferase with a broad substrate repertoire that enables it to regulate chromatin-associated processes as well as non-histone signaling proteins (Figure 1). This functional versatility is supported by a distinctive architecture shared within the SET and MYND domain-containing protein family. SMYD2 uses S-adenosylmethionine (SAM) as a methyl donor and catalyzes lysine methylation in histone and non-histone substrates. Its catalytic core contains the N-SET, SET-I, and cysteine-rich post-SET regions, while the intervening MYND zinc-finger-like domain mediates selective protein–protein interactions that contribute to substrate recognition. Completing this architecture, a C-terminal domain composed of seven twisted α-helices defines the second lobe and helps shape the deep U-shaped catalytic cleft. Together, these elements form the SAM-binding pocket, the peptide-substrate cavity, and a lysine-access channel that positions the target side chain for methyl transfer [19,20,21,22,23].

SMYD2 was initially linked to chromatin regulation through methylation of histone H3 at lysine 36, an activity associated with transcriptional repression in specific settings. Subsequent studies showed that interaction with HSP90 can alter SMYD2 substrate preference and favor H3K4 methylation, which is more commonly associated with transcriptional activation [21,25]. These findings illustrate that SMYD2 activity is shaped by interacting partners, substrate availability, and cellular context. Importantly, much of its biological impact is mediated through non-histone substrates involved in transcription, cell-cycle control, DNA repair, inflammatory signaling, metabolism, and cytoskeletal remodeling [25,26,27]. Its nuclear and cytoplasmic localization further allows SMYD2 to connect chromatin regulation with signaling pathways operating outside the nucleus. This substrate versatility explains how SMYD2 can influence multiple adaptive programs without acting through a single downstream pathway. It also provides a structural rationale for pharmacological targeting: because the peptide-binding region is less conserved than the SAM-binding pocket across lysine methyltransferases, selective inhibitors can exploit the substrate-binding cavity and lysine-access channel to achieve target specificity [17,22].

## 3. Role of SMYD2 in Cancer

Given its ability to regulate multiple cellular processes through lysine methylation, SMYD2 has emerged as an important modulator of cancer biology. Aberrant SMYD2 expression has been reported across multiple tumor types, including hepatocellular carcinoma (HCC), esophageal squamous cell carcinoma (ESCC), gastric, breast, lung, bladder, renal, and pancreatic cancers [18,25,28]. In HCC, increased SMYD2 expression has also been associated with aggressive tumor phenotypes and adverse clinical features, as discussed in detail below.

Mechanistically, SMYD2 regulates cancer-associated pathways through the methylation of both histone and, particularly, non-histone substrates involved in tumor suppressor activity, growth signaling, DNA repair, and cytoskeletal remodeling (Figure 2). One of its best-characterized substrates is p53 [29]. SMYD2 monomethylates p53 at Lys370 (p53K370me1), repressing p53-dependent transcription and thereby attenuating its tumor-suppressor activity. This mechanism represents one of several layers of p53 regulation, which also include MDM2-dependent ubiquitination modulated by CSA and CSB [30]. Similarly, SMYD2-mediated methylation of PTEN at Lys313 promotes phosphorylation at Ser380, a modification associated with reduced PTEN activity and adoption of a closed conformation, thereby favoring PI3K–AKT signaling and cancer cell growth [31]. In esophageal squamous cell carcinoma, however, SMYD2-dependent proliferation was reported to occur independently of TP53 mutation status, further illustrating the context dependence of its oncogenic effects [32]. Moreover, SMYD2 has been shown to methylate and enhance signaling through STAT3 and the p65 subunit of NF-κB, reinforcing proliferative and inflammatory programs relevant to cancer [17,33]. Conversely, methylation of the retinoblastoma protein (RB) at Lys860 generates a binding site for the methyl-lysine reader L3MBTL1, an interaction proposed to reinforce RB/E2F-dependent transcriptional repression [27]. This contrasting effect illustrates that the functional consequences of SMYD2-mediated methylation are highly substrate- and context-dependent rather than uniformly oncogenic.

Beyond proliferation and metabolic regulation, SMYD2 also participates in the cellular response to DNA damage. In non-HCC cancer models, Ku70 has been identified as a relevant non-histone substrate, with SMYD2-mediated methylation at Lys74, Lys516, and Lys539 facilitating its accumulation at DNA damage sites and the subsequent recruitment of Ku80 and DNA-PKcs. Through this mechanism, SMYD2 supports non-homologous end joining (NHEJ) and efficient repair of DNA double-strand breaks. Conversely, genetic or pharmacological inhibition of SMYD2 compromises NHEJ, leading to persistent DNA damage and accumulation of cytosolic DNA, which can activate cGAS–STING-dependent innate immune signaling [26]. Although the contribution of this pathway to HCC biology remains to be established, it provides a mechanistic basis for considering SMYD2-dependent DNA repair as a potential modulator of antitumor immunity and therapeutic response. Another dimension of SMYD2 activity involves cytoskeletal remodeling and tumor-cell plasticity. In breast cancer models, SMYD2 monomethylates BCAR3 at Lys334, creating a docking site for formin-like proteins and promoting lamellipodia dynamics, migration, and metastatic behavior [34]. While evidence for this pathway in HCC is currently lacking, these findings illustrate the broader capacity of SMYD2-dependent non-histone methylation to regulate invasive phenotypes beyond transcriptional control. These diverse activities illustrate the context-dependent biology of SMYD2 and provide a framework for examining its specific contribution to HCC [18].

## 4. SMYD2 in Hepatocellular Carcinoma

In HCC, SMYD2 has been studied in relation to tumor growth, prognosis, metabolic reprogramming, stemness, and immune regulation. Analyses of human tumor samples have demonstrated increased SMYD2 expression at both the mRNA and protein levels compared with adjacent non-tumoral liver tissue. Consistent with these findings, elevated SMYD2 expression has been associated with adverse clinicopathologic features, including larger tumor size, vascular invasion, poor histological differentiation, and advanced TNM stage. Moreover, high SMYD2 expression has been associated with shorter overall survival in a retrospective HCC cohort [35].

Beyond these clinicopathological associations, functional studies support a direct role of SMYD2 in HCC biology. SMYD2 depletion has been shown to suppress tumor cell proliferation and induce G0/G1 cell-cycle arrest, indicating a tumor cell-intrinsic contribution to HCC growth [35,36]. More recent studies have extended these observations by linking SMYD2 to metabolic reprogramming, stemness-related phenotypes, and therapeutic resistance. In particular, SMYD2 has been shown to promote glutamine metabolism through regulation of the c-Myc/GLS1 axis, providing a mechanistic link between SMYD2 activity and the metabolic requirements of HCC cells [37]. Given the marked molecular heterogeneity of HCC [38], these findings suggest that SMYD2 may contribute to tumor progression through multiple, potentially context-dependent mechanisms rather than through a single dominant pathway.

## 5. SMYD2 and Metabolic Reprogramming of HCC

Metabolic reprogramming is a hallmark of HCC, enabling tumor cells to sustain proliferation and adapt to metabolic stressors such as hypoxia and nutrient deprivation [39,40]. This adaptation involves extensive remodeling of glucose, lipid, and amino acid metabolism [39,40]. In this context, altered lysine metabolism has also been associated with immune-microenvironment remodeling and therapeutic responses in HCC [41]. These metabolic programs are coordinated by interconnected signaling pathways, including the PI3K/AKT/mTOR, Ras–Raf–MEK–ERK–c-Myc, and LKB1–AMPK pathways [39,40]. Rather than functioning as isolated metabolic circuits, these pathways form an integrated network that couples nutrient availability and cellular energy status to tumor growth and survival.

Among the HCC-specific mechanisms linking SMYD2 to metabolic reprogramming, regulation of glutamine metabolism is particularly well characterized. Glutamine supports multiple cellular functions required for tumor growth, including anaplerotic carbon supply, biosynthetic reactions, energy production, and maintenance of redox homeostasis through the generation of glutathione (GSH) and NADPH. A major regulator of this metabolic program is c-Myc, which enhances glutaminolysis and promotes expression of glutaminase 1 (GLS1), the enzyme that catalyzes the conversion of glutamine to glutamate [39,40].

Within this metabolic framework, SMYD2 has been shown to act as a post-translational regulator of c-Myc stability in HCC. SMYD2-dependent methylation stabilizes c-Myc in HCC by limiting FBW7-mediated proteasomal turnover [37]. Stabilized c-Myc, in turn, promotes GLS1 expression and enhances glutamine utilization (Figure 3). Consistent with this mechanism, SMYD2 depletion decreases c-Myc and GLS1 levels, reduces glutamine consumption and glutamate production, and suppresses HCC cell proliferation, supporting a functional role for the SMYD2–c-Myc–GLS1 axis in HCC metabolic reprogramming [37].

By sustaining c-Myc/GLS1-dependent glutaminolysis, SMYD2 may support the bioenergetic, biosynthetic, and redox requirements of proliferating HCC cells. Glutamine metabolism is closely interconnected with oncogenic signaling networks, including mTORC1 and mTORC2–AKT–c-Myc pathways, creating bidirectional interactions between metabolic state and growth signaling [40]. Increased GLS1 activity and glutamine dependence have been associated with aggressive HCC phenotypes, further supporting the biological relevance of this metabolic program. These observations position the SMYD2–c-Myc–GLS1 axis as a mechanistically defined link between lysine methylation and metabolic adaptation in HCC and suggest a potential metabolic vulnerability in SMYD2-high tumors.

## 6. SMYD2 at the Interface Between Chronic Liver Injury and the HCC Microenvironment

Most HCCs arise in chronically injured hepatic tissue rather than in an otherwise normal organ. In this setting, fibrosis, chronic inflammation, metabolic dysfunction, hypoxia, and tissue remodeling generate a permissive microenvironment that precedes and accompanies malignant transformation. This distinction is important because SMYD2 may influence HCC biology not only through tumor cell-intrinsic mechanisms, but also through effects on the diseased liver microenvironment from which tumors arise (Figure 4) [42,43].

Fibrosis represents one of the most relevant premalignant conditions in HCC development. In fibrotic liver tissue, SMYD2 has been reported to promote hepatic stellate cell (HSC) activation through regulation of the TLR4/NF-κB pathway [44]. Mechanistically, ChIP analyses showed increased SMYD2 occupancy and H3K36me2 enrichment at the Tlr4 promoter in activated HSCs, consistent with enhanced Tlr4 transcription (Figure 3). SMYD2 silencing also reduces TGF-β1-induced HSC activation together with decreased expression of TLR4, NF-κB signaling components, type I collagen, and α-smooth muscle actin (α-SMA). These findings suggest that SMYD2 may contribute to the establishment of a fibrotic and inflammatory milieu permissive for hepatocarcinogenesis, although they do not establish a direct role in hepatocyte transformation. These observations are also consistent with a broader role of SMYD2 in inflammatory signaling. In other disease contexts, SMYD2 has been shown to methylate TRAF2, limiting its proteolytic degradation and thereby sustaining NF-κB activation [45] (Figure 3). This mechanism raises the possibility that SMYD2 may amplify inflammatory signaling beyond HSCs; however, whether TRAF2-dependent SMYD2 signaling operates in chronic liver disease or HCC-associated inflammation remains to be established.

SMYD2 has also been implicated in metabolic liver disease, an increasingly important etiological background for HCC. In models of diet-induced metabolic stress, in vivo CRISPR-based screening identified Smyd2 loss as a fitness-promoting alteration in hepatocytes, and liver-wide Smyd2 deletion reduced hepatic steatosis and liver injury [46]. Transcriptomic analyses showed reduced expression of genes involved in fatty acid and triglyceride synthesis together with increased expression of TCA-cycle genes, consistent with a shift away from lipid storage and toward oxidative metabolism. Pharmacological inhibition with AZ-505 similarly reduced steatosis and liver triglyceride accumulation in mice exposed to a Western diet, although in this study treatment was initiated concomitantly with the dietary challenge and therefore primarily modeled prevention or early disease modification rather than reversal of established MASH [46]. Independent transcriptomic analyses of human liver samples have also identified SMYD2 among the epigenetic regulators upregulated in NASH/MASH compared with normal liver, supporting an association between SMYD2 dysregulation and metabolic liver disease [47]. However, this interpretation requires caution. In a distinct choline-deficient, L-amino acid-defined, high-fat diet (CDAHFD) model of established steatohepatitis, treatment with AZ-505 or LLY-507 failed to improve NASH activity scores, liver injury, fibrosis, macrophage infiltration, or hepatic inflammation [48]. These divergent findings suggest that the effects of SMYD2 inhibition in metabolic liver disease are highly model- and stage-dependent, with stronger evidence for modulation of steatosis and early metabolic adaptation than for reversal of established inflammatory and fibrotic disease. Accordingly, the contribution of SMYD2 to MASLD/MASH progression, and particularly to MASLD-associated HCC, remains incompletely defined.

## 7. SMYD2 at the Tumor–Immune Interface in HCC

Beyond its tumor cell-intrinsic functions, SMYD2 has also been implicated in the regulation of immune suppression in HCC. The most direct evidence comes from experimental HCC models showing that SMYD2 activity in tumor cells is associated with increased expression of Wnt ligands, including Wnt3a and Wnt5a, and with paracrine effects on surrounding macrophages that favor a suppressive phenotype characterized by increased TGF-β signaling [49].

Conversely, in experimental HCC, pharmacological SMYD2 blockade altered tumor-derived Wnt signaling and attenuated the TGF-β-associated macrophage response, suggesting that SMYD2 contributes to tumor–myeloid crosstalk within the immunosuppressive microenvironment [49]. This Wnt/TGF-β-enriched microenvironment may suppress effector T-cell activity and reinforce immune tolerance, providing a mechanistic link between SMYD2 activity, Wnt-associated tumor–macrophage crosstalk, and resistance to antitumor immunity [49].

This mechanism is consistent with a broader relationship between SMYD2 and Wnt/β-catenin signaling. SMYD2-mediated methylation of β-catenin at Lys133 has been shown to facilitate its nuclear translocation and transcriptional activity, including in HCC cell lines [50] (Figure 3). Thus, SMYD2 may reinforce Wnt signaling at two complementary levels: by promoting β-catenin activity within tumor cells and by enhancing Wnt ligand-dependent communication with macrophages in the tumor microenvironment [49,50,51].

SMYD2 may also influence antitumor immunity through its role in DNA damage repair. In non-HCC models, SMYD2 inhibition can link impaired DNA repair to cGAS–STING-dependent innate immune signaling [26]. Whether this mechanism contributes to immune regulation in HCC remains to be established. Bioinformatic analyses in HCC showing an inverse association between SMYD2 expression and activated CD8+ T-cell abundance are consistent with an immunosuppressive role for SMYD2, but they do not establish that the DNA damage–cGAS–STING pathway mediates this association in liver tumors [49].

The effects of SMYD2 on inflammatory signaling appear to depend on the cellular compartment. While SMYD2 has been associated with pro-inflammatory signaling in hepatic stellate cells and other disease models (Section 6) [44,45], its activity in macrophages may have opposing effects. In mouse bone marrow-derived macrophages, SMYD2 overexpression reduced pro-inflammatory cytokine production and shifted T-cell differentiation away from Th17 and toward regulatory T-cell phenotypes, accompanied by increased TGF-β and reduced IL-6 production (Figure 3). Mechanistically, SMYD2 increased H3K36 dimethylation at the Tnf and Il6 promoters and suppressed their transcription [52].

These divergent observations indicate that SMYD2 should not be classified as uniformly proinflammatory or anti-inflammatory. Rather, its biological output appears to depend on the cellular compartment, disease context, substrate availability, and local cytokine environment. For HCC, this distinction is particularly important because SMYD2 activity in tumor cells, macrophages, hepatic stellate cells, and non-tumoral hepatocytes may exert distinct, and potentially opposing, effects on tumor progression and antitumor immunity [44,49,52]. Understanding how these effects vary across etiological and molecular subtypes of HCC will be essential for assessing the therapeutic potential of SMYD2 inhibition (Table 1).

## 8. SMYD2 as a Therapeutic Target

Accumulating evidence that SMYD2 contributes to tumor progression, together with the identification of SMYD2-dependent vulnerabilities, has stimulated interest in targeting this methyltransferase therapeutically. Structural characterization of the enzyme was critical for translating this biological rationale into selective pharmacological strategies. Because the substrate-binding region is generally less conserved among protein lysine methyltransferases than the SAM-binding pocket, medicinal chemistry efforts have largely focused on the peptide-binding site, leading predominantly to the development of substrate-competitive inhibitors [23,55,56].

In this context, AZ-505 was the first selective SMYD2 inhibitor reported and provided proof of concept that the enzyme could be pharmacologically targeted with selectivity over other lysine methyltransferases. AZ-505 acts as a substrate-competitive inhibitor by binding within the peptide-binding groove and partially overlapping the substrate-binding site (Figure 5), thereby preventing productive substrate recognition [23]. Subsequent medicinal chemistry efforts led to the development of compounds such as LLY-507, A-893, and BAY-598, which retained a substrate-competitive mechanism while achieving improved biochemical potency, cellular activity, and/or selectivity through optimized interactions within the peptide-binding cavity. LLY-507 contains a pyrrolidine group that occupies the substrate lysine-binding channel, whereas BAY-598 inserts a chlorophenyl moiety into the same channel and establishes additional interactions within the peptide-binding pocket (Figure 5) [56,57].

More recently, EPZ033294 and the structurally related EPZ032597 expanded the pharmacological repertoire of SMYD2 inhibitors by introducing a peptide-substrate-noncompetitive mechanism of inhibition. EPZ033294 inhibits SMYD2 with low-nanomolar biochemical potency and is noncompetitive with respect to the peptide substrate, while its inhibition relative to SAM has been characterized as noncompetitive or uncompetitive [58]. Structural analysis revealed an unusual binding configuration in which EPZ033294 extends across the peptide-recognition region and remodels the local SMYD2 structure to create a hydrophobic cavity that accommodates the distal portion of the inhibitor (Figure 5). The related compound EPZ032597 similarly displays noncompetitive inhibition with respect to the peptide substrate [58], further expanding the range of SMYD2 inhibitors with distinct pharmacological properties and levels of preclinical characterization (Table 2).

Preclinical studies in selected tumor models indicate that genetic or pharmacological SMYD2 inhibition can suppress tumor growth, although this effect is not uniform across all cancer contexts [17,18,61]. In contrast, highly selective SMYD2 inhibitors and CRISPR/Cas9-mediated gene disruption failed to impair autonomous proliferation across large panels of cancer cell lines [58]. These findings highlight the importance of distinguishing between catalytic inhibition, genetic disruption, and their effects on cell proliferation, as these approaches and outcomes do not necessarily provide equivalent evidence of therapeutic vulnerability. Furthermore, active-site inhibitors may not affect potential non-catalytic functions of SMYD2, although their relevance to HCC remains to be established.

In HCC, functional studies support a role for SMYD2 in tumor growth, metabolic adaptation, stemness, and therapeutic resistance. SMYD2 knockdown suppresses HCC cell proliferation and induces G0/G1 arrest, whereas disruption of the SMYD2–c-Myc–GLS1 axis reduces glutamine-dependent metabolic adaptation [35,37,53]. More recently, SMYD2 was shown to contribute to sorafenib resistance by maintaining cancer stem cell properties through epigenetic activation of the BMP4/R-SMADs/ID3 axis (Figure 3). Consistent with this mechanism, SMYD2 knockdown or pharmacological inhibition reduced BMP4 expression, R-SMAD activation, ID3 expression, stemness, and sorafenib resistance, whereas restoration of BMP4 or ID3 partially rescued these phenotypes [53].

Evidence from other malignancies suggests that combination strategies may also extend to cytotoxic agents. In non-small-cell lung cancer, the SMYD2 inhibitor BAY-598 enhanced cisplatin sensitivity through reactivation of the p53 pathway, increasing p21, GADD45, and Bax expression and promoting apoptosis [62]. A separate study showed that BAY-598 synergized with doxorubicin in NSCLC models, with combined treatment suppressing tumor growth, migration, and invasion and attenuating JAK/STAT signaling [63].

Although these findings support a broader role for SMYD2 in treatment adaptation, their relevance to HCC beyond sorafenib remains to be established. In HCC, targeting other epigenetic regulators, such as LSD1, has been shown to reshape the tumor microenvironment and overcome acquired resistance to anti-PD-1 therapy [64]. Similarly, pharmacological inhibition of SMYD2 with AZ-505 enhanced the antitumor response to anti-PD-1 therapy in preclinical HCC models, producing greater tumor control than either treatment alone. This effect was particularly evident in tumors with activated Wnt/β-catenin signaling, in which SMYD2 contributes to an immunosuppressive microenvironment through Wnt-dependent tumor–macrophage communication and TGF-β-associated signaling [49].

Targeting DNA repair mechanisms represents another potential strategy to enhance therapeutic sensitivity. This approach has been explored in melanoma, where ERCC8/CSA silencing increased sensitivity to alkylating agents such as dacarbazine and temozolomide [65]. Through a distinct mechanism, SMYD2 inhibition may also enhance sensitivity to DNA-damaging treatments. In preclinical models outside HCC, genetic or pharmacological SMYD2 inhibition impairs DNA double-strand break repair and promotes innate immune signaling [26]. These findings provide a rationale for combining SMYD2 inhibition with radiotherapy and potentially with immune-based therapies, although this strategy remains to be validated in HCC-specific models.

Overall, the available evidence favors evaluating SMYD2 inhibition as part of mechanism-based combination strategies rather than assuming broad efficacy as monotherapy. The benefit of each combination is likely to depend on tumor type, molecular context, and the specific SMYD2-dependent pathway driving treatment resistance. This context dependence is particularly important given that potent pharmacological inhibition or genetic ablation of SMYD2 does not uniformly impair cancer-cell proliferation across experimental systems [58].

## 9. Knowledge Gaps and Future Directions

Despite progress in defining the functions of SMYD2 in HCC, substantial biological and translational uncertainties continue to limit its clinical development. Most mechanistic evidence derives from established cell lines, xenografts, and murine models, whereas associations between elevated SMYD2 expression, aggressive clinicopathological features, and poor clinical outcomes are largely based on retrospective analyses of patient samples and transcriptomic datasets. These observations require validation in independent, clinically annotated cohorts to establish their robustness and clinical relevance.

Beyond the need for clinical validation, several SMYD2-dependent mechanisms have been characterized in HCC. However, the evidence supporting individual pathways, including glutamine metabolism, stemness-associated programs, and tumor–macrophage communication, derives largely from individual studies. Independent validation across additional HCC models and molecular backgrounds is needed. Moreover, no SMYD2 inhibitor has entered clinical evaluation in HCC, leaving its pharmacokinetic properties, safety profile, therapeutic window, and degree of target engagement in tumor and non-tumoral liver unresolved.

These limitations of current experimental systems are particularly relevant because HCC usually develops within chronically diseased liver rather than in otherwise normal tissue. Conventional cell-line and xenograft models incompletely reproduce the inflammatory, fibrotic, metabolic, vascular, and immune alterations associated with cirrhosis and other etiological backgrounds. Dependence on SMYD2 is also unlikely to be uniform across the molecular diversity of HCC. Available evidence suggests that SMYD2 activity may be particularly relevant in tumors characterized by stemness-associated programs, altered glutamine metabolism, Wnt/β-catenin signaling, or therapeutic resistance, but the molecular determinants of this dependency remain poorly defined. The influence of disease etiology and cellular compartment also requires clarification, as SMYD2 activity in tumor cells, hepatocytes, hepatic stellate cells, and immune populations may produce distinct or even opposing biological effects. This context dependence is further illustrated by the selection of Smyd2-deficient hepatocyte clones during metabolic liver injury [46], the lack of benefit from SMYD2 inhibitors in established steatohepatitis [48], and the antitumor effects observed in experimental HCC [49]. Importantly, these studies involve distinct disease settings, interventions, and biological endpoints, limiting direct comparisons between their findings.

A further translational consideration is that SMYD2 may exert protective effects in non-tumoral liver under acute stress. In hepatic ischemia–reperfusion models, increased SMYD2 activity was associated with reduced hepatocyte inflammation and apoptosis, with mechanistic experiments identifying STAT1 Lys175 methylation as a critical determinant of this protective effect [54]. Consistent with these experimental findings, higher post-I/R SMYD2 expression in human hepatectomy samples correlated with lower postoperative ALT and AST levels and better biochemical recovery [54]. This apparent contrast between hepatoprotection in injured liver and tumor-promoting SMYD2 activity in HCC emphasizes the strong dependence of SMYD2 function on cellular and disease context [66].

Although these findings do not establish that systemic SMYD2 inhibition would impair liver recovery in patients with HCC, they raise a potential concern for its therapeutic use. Clinical development will therefore require careful evaluation of the balance between antitumor activity and preservation of normal hepatic function, particularly in patients with cirrhosis, limited hepatic reserve, or recent liver injury. This issue may become especially relevant in clinical settings involving liver resection or other interventions in which regenerative capacity and resistance to ischemic injury are critical.

Future studies should therefore evaluate antitumor efficacy and hepatic toxicity simultaneously in immunocompetent, etiology-relevant models that preserve the diseased liver microenvironment. Pharmacological development should prioritize mechanism-based combinations for which direct preclinical evidence is available in HCC, including immune checkpoint blockade and selected targeted therapies such as sorafenib, whereas strategies involving radiotherapy or other treatment modalities require HCC-specific validation. These studies should incorporate pharmacodynamic measurements of SMYD2 inhibition, assessment of target engagement in both tumor and non-tumoral liver, and molecular criteria capable of identifying tumors in which a specific SMYD2-dependent pathway contributes to therapeutic resistance. To this end, exploratory stratification could combine SMYD2 expression with functional substrate-methylation readouts, Wnt/β-catenin activity, c-Myc/GLS1-associated glutamine-metabolism signatures, and macrophage/TGF-β/CD8 immune profiles. None of these candidate readouts has been prospectively validated to predict response to SMYD2 inhibition in HCC.

Such an approach will be necessary to distinguish SMYD2 expression from therapeutically actionable SMYD2 dependency, to identify the biological contexts most likely to respond to inhibition, and ultimately to determine whether sufficient tumor selectivity and therapeutic window can be achieved for clinical translation.

## 10. Conclusions

SMYD2 regulates multiple processes implicated in HCC progression through the methylation of both histone and non-histone substrates. Current evidence links its activity to metabolic adaptation, stem-like tumor phenotypes, DNA damage repair, therapeutic resistance, and communication between tumor cells and components of the hepatic microenvironment. Its biological relevance may therefore lie not in a single downstream pathway, but in the context-dependent convergence of tumor-intrinsic programs with the inflammatory, fibrotic, metabolic, and immune alterations that characterize chronically diseased liver.

However, the available evidence remains predominantly preclinical, and neither the uniform dependence of HCC on SMYD2 nor a clinical benefit from its inhibition has yet been established. Defining the molecular and etiological contexts in which SMYD2 is functionally required, identifying the cellular compartments that mediate therapeutic responses, and determining the consequences of SMYD2 inhibition in non-tumoral liver will be essential before this enzyme can be confidently advanced as a therapeutic target.

At present, SMYD2 is best regarded as a mechanistically relevant and potentially actionable vulnerability whose translational value will depend on appropriate biological stratification, the demonstration of a sufficiently favorable therapeutic window, and the development of rational combination strategies tailored to the specific SMYD2-dependent mechanisms operating in individual HCC contexts.

## Figures and Tables

**Figure 1:**
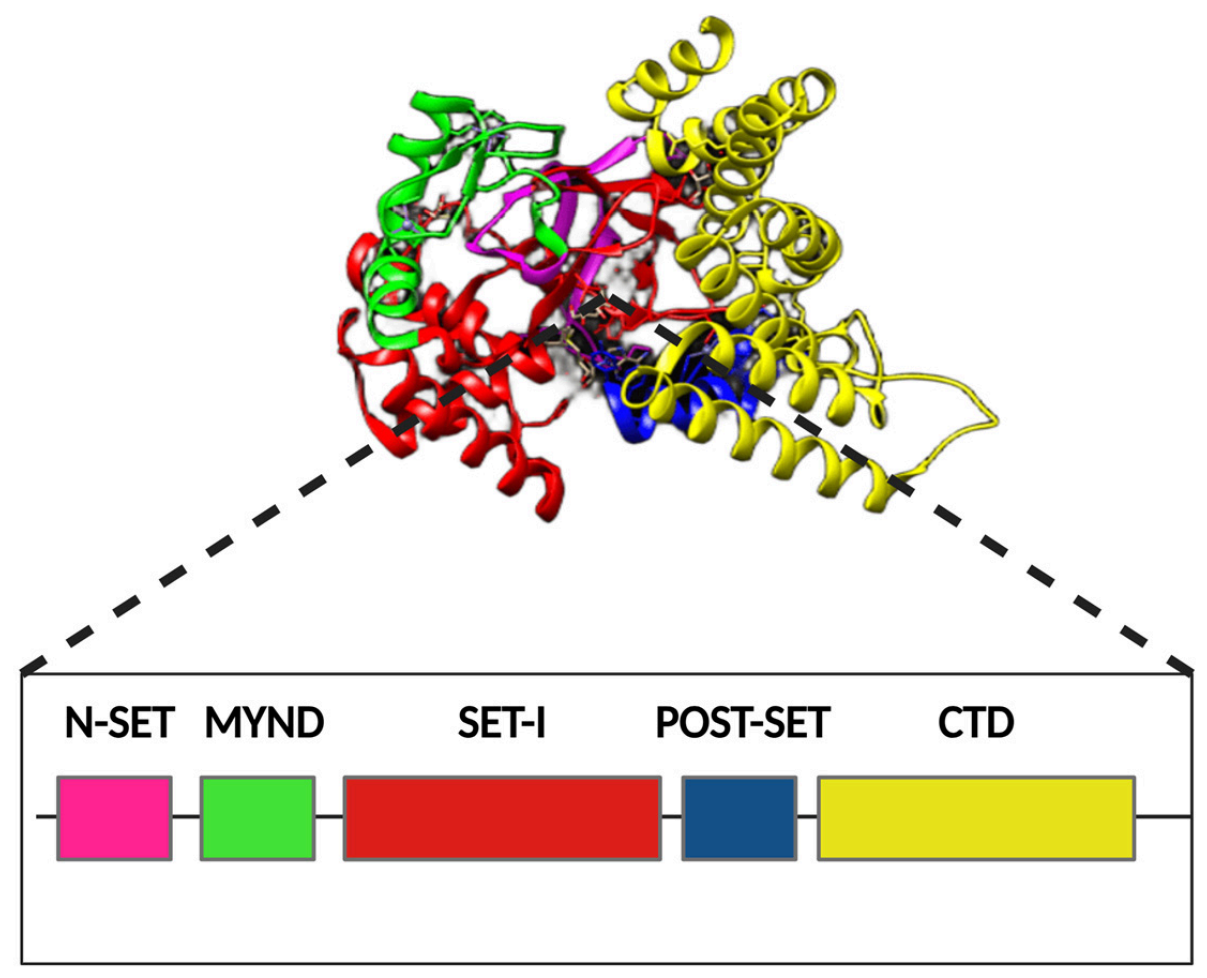
Structural organization and domain architecture of SMYD2. Schematic representation of the three-dimensional structure and linear domain organization of SMYD2. The protein contains N-SET, MYND, SET-I, post-SET, and C-terminal domain (CTD) regions, which together contribute to substrate recognition and formation of the catalytic cleft. The three-dimensional structure was obtained from the Protein Data Bank (PDB ID: 3TG4 [24]), and the different domains were color-coded using UCSF Chimera.

**Figure 2:**
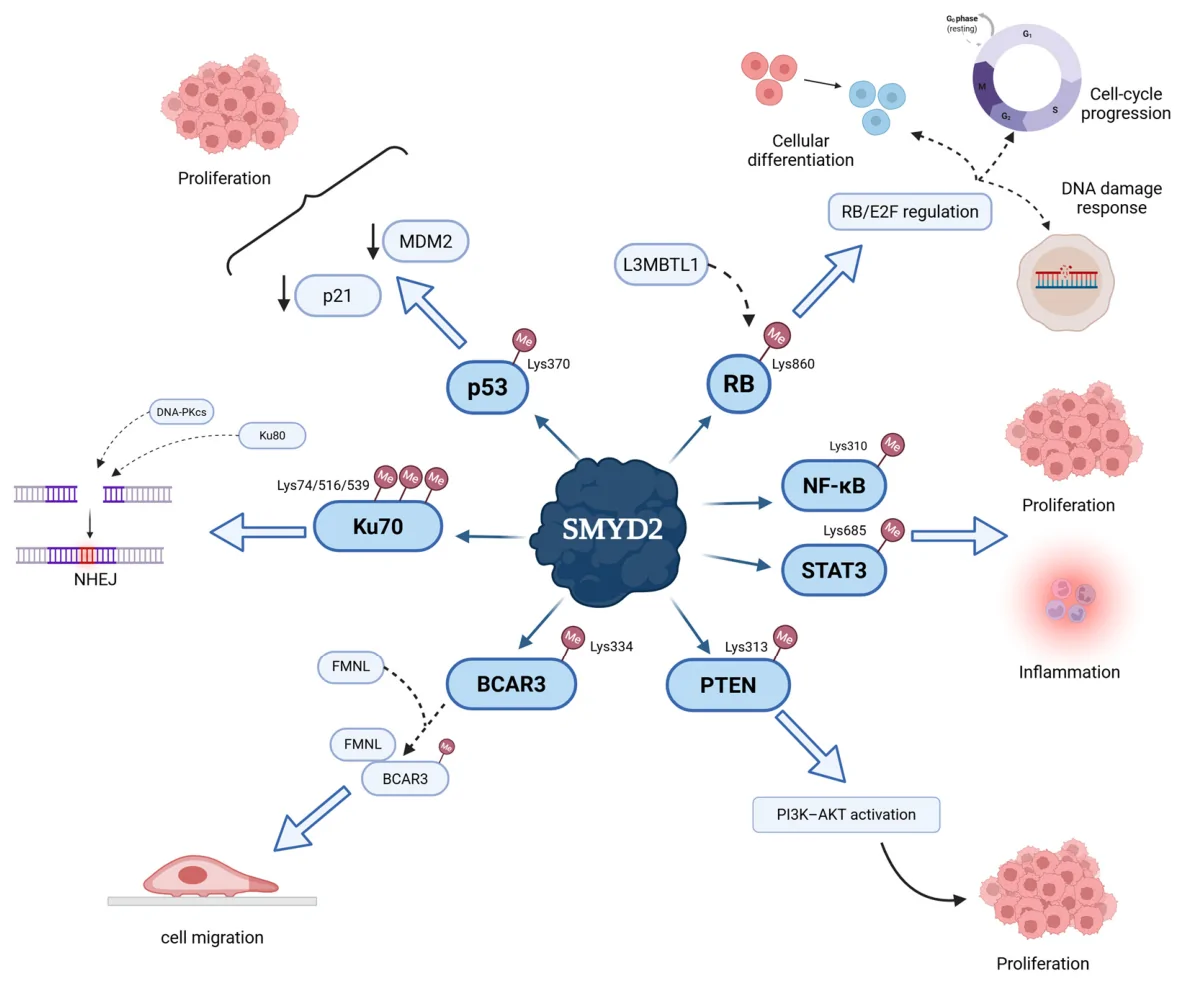
Non-histone substrates and functional outputs of SMYD2-mediated methylation. Schematic overview of non-histone SMYD2 substrates and their functional effects, characterized predominantly in experimental models outside HCC. Representative substrates include p53, RB, Ku70, BCAR3, PTEN, NF-κB, and STAT3, which participate in tumor suppressor regulation, DNA damage response, inflammatory signaling, cytoskeletal remodeling, and growth-associated pathways. SMYD2-mediated methylation of these substrates has been linked to effects on p53-dependent transcription, RB/E2F regulation, non-homologous end joining (NHEJ), cell migration, PI3K–AKT activation, inflammation, and proliferation.

**Figure 3:**
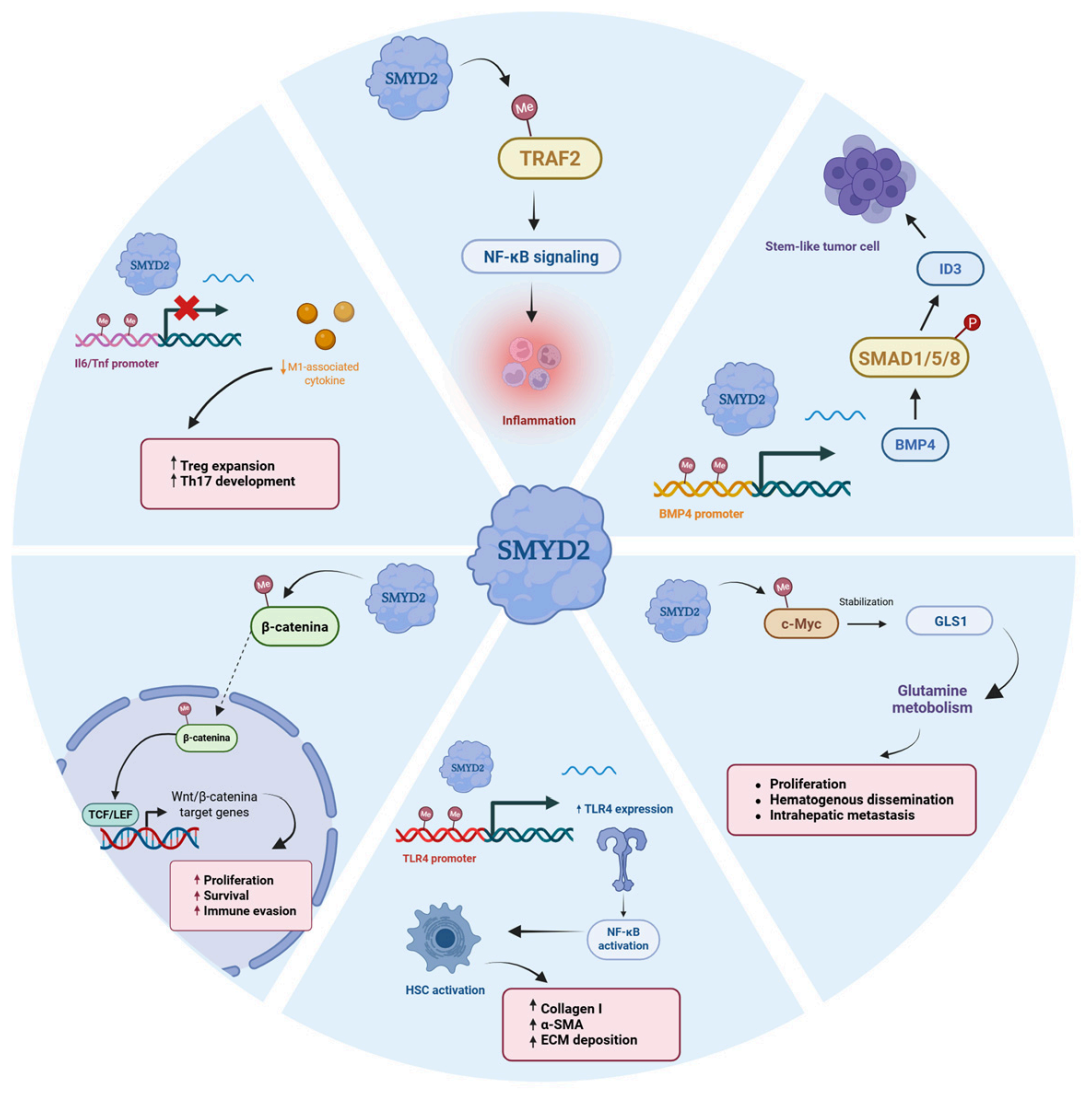
SMYD2-dependent pathways implicated in HCC-relevant biology. Schematic overview of SMYD2-associated pathways in tumor, stromal, and immune compartments, based on evidence from HCC and other experimental models. SMYD2 has been linked to β-catenin methylation and Wnt/β-catenin target gene activation, c-Myc stabilization and GLS1-dependent glutamine metabolism, BMP4/SMAD1/5/8/ID3-associated stemness programs, TLR4/NF-κB-dependent hepatic stellate cell activation, TRAF2/NF-κB inflammatory signaling, and macrophage-intrinsic regulation of Tnf and Il6 transcription. These pathways illustrate the compartment-specific biology of SMYD2 and its potentially divergent effects across tumor cells, stromal cells, and immune populations.

**Figure 4:**
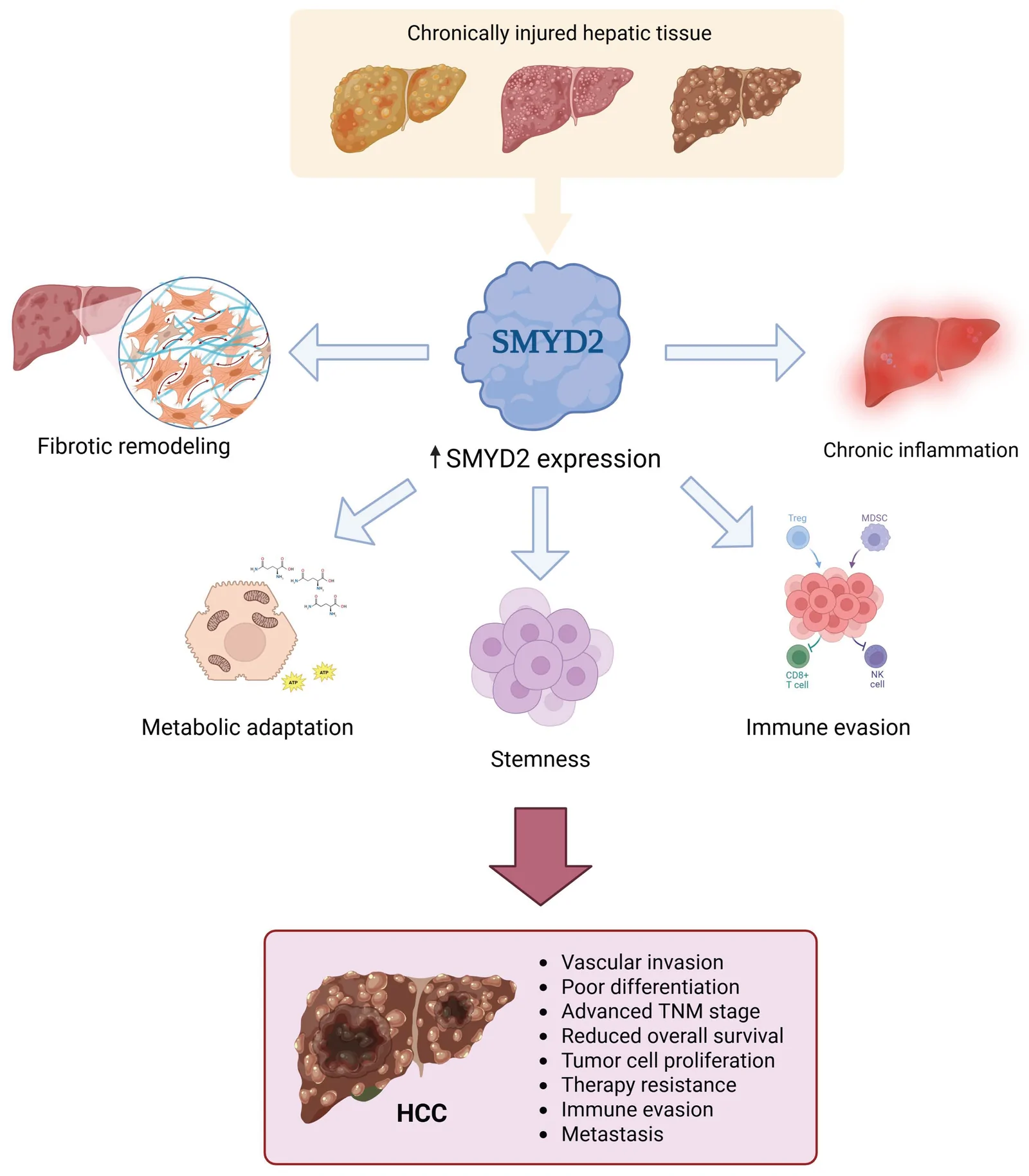
SMYD2 dysregulation in the diseased liver microenvironment and HCC progression. Conceptual model integrating associations between SMYD2 expression, alterations in the hepatic microenvironment, and HCC progression, based on evidence from different experimental and clinical settings. In chronically injured hepatic tissue, SMYD2 has been associated with fibrotic remodeling, chronic inflammation, metabolic adaptation, stemness-associated programs, and immune evasion. These processes may contribute to aggressive HCC features, including vascular invasion, poor differentiation, advanced TNM stage, reduced overall survival, tumor cell proliferation, therapy resistance, immune escape, and metastasis.

**Figure 5:**
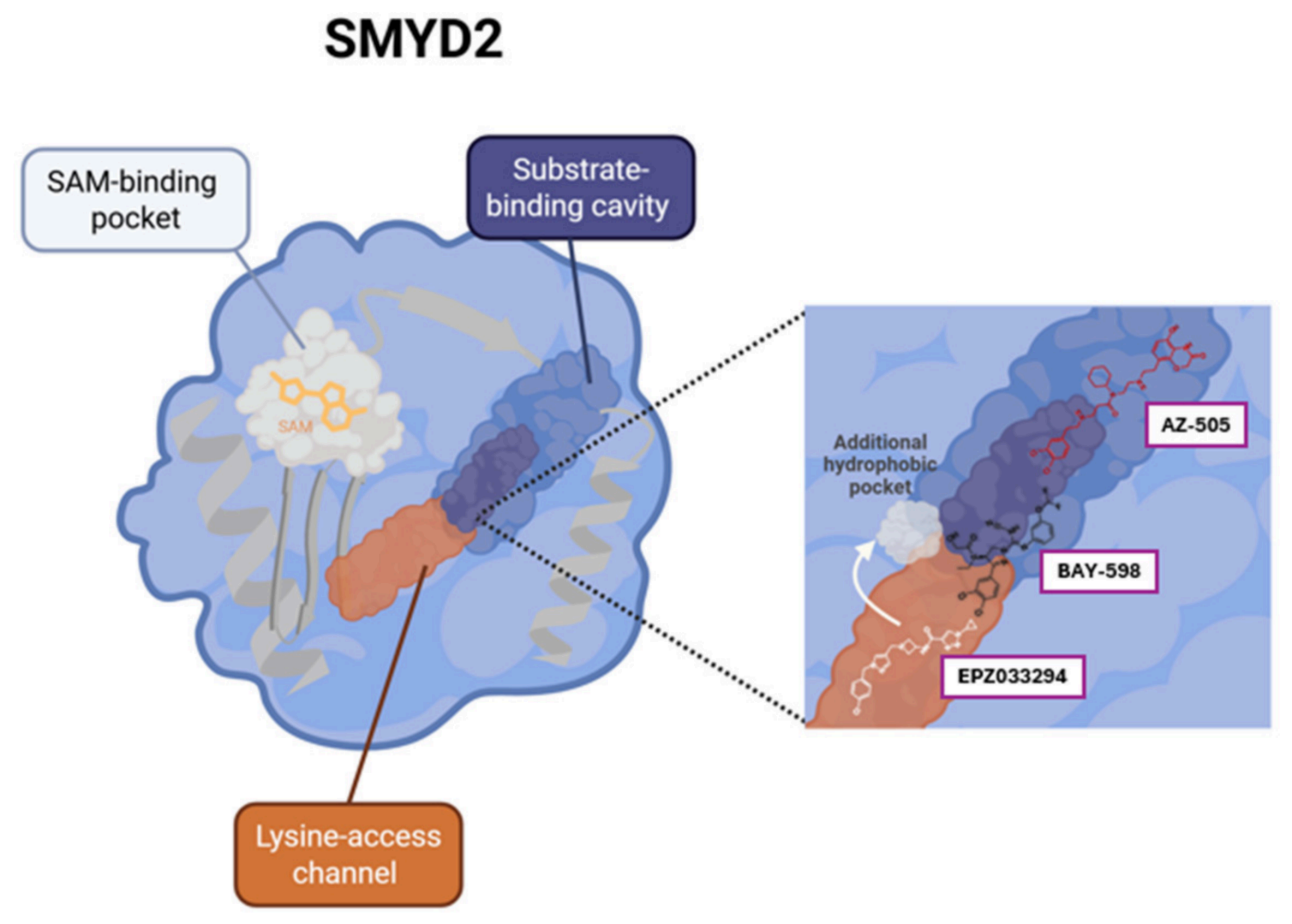
Structural basis for pharmacological targeting of SMYD2. Schematic representation of the main druggable regions within SMYD2, including the S-adenosylmethionine (SAM)-binding pocket, substrate-binding cavity, and lysine-access channel. Representative inhibitors occupy or remodel regions of the substrate-recognition surface, as illustrated for AZ-505, BAY-598, and EPZ033294. These binding modes provide a structural rationale for selective SMYD2 inhibition through regions that are less conserved than the SAM-binding pocket. The protein structure is a conceptual representation and does not correspond to an experimental crystallographic structure. The chemical structures of the inhibitors (AZ-505, BAY-598, and EPZ033294) are based on their respective reported molecular structures.

**Table 1:** Experimental evidence for SMYD2-associated mechanisms in HCC and related contexts.

SMYD2 Mechanism/Finding	Experimental Model	HCC Evidence	Ref.
Clinical prognosis	163 HCC cases; IHC; cell lines	Yes	[35]
p53 K370 methylation	Biochemical; cell assays	No	[29]
PTEN K313 methylation	Biochemical; non-HCC cells	No	[31]
BCAR3 K334/migration	Breast cancer cells; mice	No	[34]
c-Myc/GLS1 metabolism	HCC tissues, cells; tumor models	Yes	[37]
BMP4/SMAD/ID3; sorafenib	Resistant HCC cells; xenografts	Yes	[53]
β-Catenin methylation/Wnt	Biochemical; cells incl. HCC	Partial	[50]
Wnt/macrophages/TGF-β; PD-1	HCC cells; macrophages; orthotopic mice	Yes	[49]
Ku70/NHEJ/cGAS–STING	Non-HCC cancer cells; mice	No	[26]
HSC/TLR4/NF-κB	Stellate cells; fibrotic mice	No (fibrosis)	[44]
Macrophage Tnf/Il6	Murine BMDMs; T-cell assays	No	[52]
Smyd2 loss/metabolic liver	CRISPR screening; diet-fed mice	No (MASLD)	[46]
AZ-505/LLY-507 in MASH	CDAHFD mice	No (MASH)	[48]
STAT1 K175/I/R protection	Mouse I/R; human hepatectomy	No	[54]

**Table 2:** Pharmacological characteristics of SMYD2 inhibitors.

Inhibitor	IC_50_ Reported	Selectivity	Cellular Target Engagement	In Vivo/PK	HCC Evidence	Refs.
AZ-505	120 nM, 51 nM	~700-fold vs. 6 KMTs	BTF3me1 IC_50_: 13.9 µM	HCC mice; PK NR	Cells; orthotopic models	[23,49,58]
LLY-507	<15 nM, 17 nM	>100-fold vs. tested KMTs	BTF3me1 IC_50_: 0.691 µM	HCC mice; PK NR	Cells; orthotopic models	[49,58,59]
BAY-598	27 nM	>100-fold vs. 31 KMTs	AHNAK methylation: ~60 nM	In vivo probe; PK reported	NR	[57]
A-893	2.8 nM	30 KMTs: no effect at 1 µM	p53K370me1: −42% at 10 µM	PK/in vivo NR	NR	[60]
EPZ033294	3.9 nM	15 KMTs: no effect at 10 µM	BTF3me1 IC_50_: 2.9 nM	PK/in vivo NR	NR	[58]
EPZ032597	16 nM	15 KMTs: no effect at 10 µM	BTF3me1 IC_50_: 31 nM	PK/in vivo NR	NR	[58]

NR, not reported; KMT, lysine methyltransferase; PK, pharmacokinetic.

## Data Availability

No new data were created or analyzed in this study. Data sharing is not applicable to this article.
